# Emerging insights into the role of visceral adipose tissue in diabetic kidney disease

**DOI:** 10.3389/fnut.2025.1715701

**Published:** 2025-11-28

**Authors:** Linrong Li, Cuiping Liu, Junling Gu

**Affiliations:** 1 North Sichuan Medical College, Nanchong, Sichuan, China; 2 Department of Endocrinology, The Second People’s Hospital of Yibin City-West China Yibin Hospital, Sichuan University, Yibin, Sichuan, China

**Keywords:** visceral adipose tissue, visceral fat area, diabetic kidney disease, central obesity, renal sinus fat

## Abstract

Diabetic kidney disease (DKD) is one of the primary microvascular complications of diabetes mellitus and the leading cause of end-stage renal disease. Obesity, which increases the risk of metabolic disorders, plays a crucial role in the onset and progression of DKD. Visceral fat area (VFA), recognized as the gold standard for diagnosing central obesity, can be accurately measured via imaging techniques. Visceral adipose tissue (VAT) regulates disease progression through a variety of potential mechanisms and promotes the occurrence and development of DKD. This review summarizes the assessment methods of VFA, the association between VAT and DKD, and the potential mechanisms by which VAT drives DKD pathogenesis, aiming to provide insights into the role of VAT assessment and its mechanisms in the prevention and treatment of DKD.

## 1 Introduction

Diabetic kidney disease (DKD) is a major chronic complication of diabetes mellitus and the leading cause of end-stage kidney disease (ESKD) (1). Epidemiological data show that approximately one-third of diabetic patients develop chronic kidney disease (CKD) (2), and 20–40% of them progress to DKD (3), imposing a heavy economic and medical burden on individuals and societies. However, the pathogenesis of DKD is complex, involving numerous risk factors and limited treatment options. Therefore, early intervention to address modifiable risk factors is crucial.

In recent years, studies have shown that central obesity characterized by visceral adipose tissue (VAT) accumulation is a key driver of the progression of DKD (4, 5). As the core pathological mediator linking metabolic disorders to renal damage, VAT urgently requires prioritized research. According to the 2025 World Obesity Report, by 2030, nearly 3 billion adults globally (accounting for approximately 50% of the global adult population) will be affected by overweight or obesity (6). The continuous surge in the global obesity rate underscores the urgency of targeting VAT. China, which has the largest obese population globally, is projected to see the prevalence of adult overweight or obesity reach 65.3% by 2030 (7). Notably, research data involving nearly 16 million people in China reveal that among overweight/obese individuals, the proportions with prediabetes are 30.7 and 36.9% (8), suggesting a strong link between adiposity and glucose metabolism disorders, which in turn contribute to DKD.

Visceral fat area (VFA), a key parameter reflecting VAT accumulation, is recognized as the gold standard for diagnosing central obesity. Existing reviews on VAT have covered its assessment methods, predictive value for diabetes and chronic complications, and associations with metabolic diseases such as type 2 diabetes mellitus (T2DM) and cardiovascular disease (9, 10). For instance, some literature indicates that the visceral adiposity index (VAI) is associated with the incidence of nephropathy in T2DM patients (11). However, systematic descriptions of the correlation between VAT (assessed by VFA or other indicators) and DKD development risk are still lacking, highlighting the need to clarify the role of VAT in DKD pathogenesis. Furthermore, in the emerging field of renal ectopic fat research in recent years, there is a paucity of studies exploring the impact of renal ectopic fat deposition on DKD progression and its predictive value. Overall, domestic and international literature on the association between VAT and DKD lacks systematic and comprehensive synthesis.

In this review, we overview the harms of central obesity and its mainstream assessment methods (with a focus on VFA). We then discuss the correlation between VAT and DKD progression, as well as the potential underlying mechanisms. Additionally, we analyze emerging therapeutic strategies targeting VAT to identify potential therapeutic targets. This synthesis aims to provide a new perspective for DKD prevention and treatment, with the goal of facilitating early identification and intervention of VAT related risk factors.

## 2 The hazards of central obesity

Obesity, characterized by excessive adipose tissue accumulation, is closely associated with a spectrum of complications that severely impair physical and mental health. These include cardiovascular diseases, diabetes mellitus, malignancies, neurological disorders, respiratory diseases, and gastrointestinal diseases, as well as non-specific issues such as sleep disturbances, mobility limitations, and psychological distress (12). A real-world cross-sectional study encompassing 15.8 million Chinese adults pinpointed the five most prevalent complications in overweight and obese individuals: fatty liver disease (49.0 and 81.8%), prediabetes (30.7 and 36.9%), dyslipidemia (31.3 and 42.4%), and hypertension (20.7 and 36.9%) (8). Beyond these, obesity is linked to cognitive dysfunction (13, 14) and elevated malignancy risk—ranking as the second leading preventable cause of cancer, with strong associations with breast, colorectal, and pancreatic cancers (15, 16). In pediatric populations, a meta-analysis of 962 obese adolescents (10–19 years old) with obstructive sleep apnea (OSA) showed that multidisciplinary weight-loss interventions significantly reduced OSA severity and prevalence while improving overall sleep quality, highlighting the tight link between pediatric obesity, OSA, and sleep deprivation (17). Additionally, obesity exacerbates reproductive complications in polycystic ovary syndrome (18) and increases susceptibility to stress urinary incontinence (19).

### 2.1 Classification of obesity and characteristics of central obesity

Obesity is categorized into peripheral obesity and central obesity based on adipose tissue distribution, which corresponds to differences in subcutaneous adipose tissue (SAT) and VAT accumulation. SAT accounts for approximately 80% of total body fat, while VAT constitutes 5–20%—the latter is primarily located in the abdominal cavity, surrounding visceral organs (e.g., mesenteric adipose tissue, perirenal adipose tissue [PRAT]) (20). Notably, excessive VAT accumulation poses far greater health risks than SAT, making central obesity (dominated by VAT excess) a key focus of clinical concern.

In China, central obesity is defined as VFA ≥ 80 cm^2^ (21), while waist circumference—an indirect measure of abdominal fat accumulation—is widely used in clinical practice, with diagnostic thresholds of ≥ 90 cm for men and ≥ 85 cm for women (22). These standards reflect ethnic differences in body composition: East Asians tend to have central obesity (VAT-dominant), whereas Europeans and Americans are more prone to generalized obesity (23). Consequently, China’s waist circumference cutoff for central obesity is lower than that of Europe (men ≥102 cm, women ≥88 cm) (24).

### 2.2 Health hazards of central obesity

Central obesity is the core component of metabolic syndrome and a pivotal link of its pathogenesis, which will lead to insulin resistance (IR), hypertension, and dyslipidemia, thus increasing the risk of metabolic diseases such as atherosclerotic cardiovascular disease (ASCVD) and T2DM (25). Epidemiological studies have confirmed that central obesity is independently associated with atherosclerosis (26) and atrial fibrillation (27), further emphasizing its cardiovascular risks.

Compared with SAT, VAT exhibits higher metabolic activity, secreting a variety of pro-inflammatory cytokines and adipokines that exacerbate IR-related metabolic disorders. This makes VAT a key driver of T2DM, atherosclerosis, and cardiovascular diseases (28, 29), and an independent risk factor for metabolic diseases in Asian populations (30). Notably, Asians have significantly higher VAT content than Caucasians at the same Body Mass Index (BMI) level, contributing to their higher susceptibility to metabolic diseases (31). The clinical significance of VAT assessment is further supported by a large-scale cohort study of 11,120 participants: the metabolic score for VAT (METS-VF, a surrogate indicator of VAT burden) showed a significant positive association with all-cause mortality, cardiovascular mortality, and cancer mortality (32).

Beyond metabolic consequences, VAT accumulation directly impacts neurological function. A Singaporean cohort study of 8,769 participants established a causal relationship between increased VAT and cognitive decline: every 0.27 kg increase in VAT mass was associated with cognitive impairment equivalent to 0.7 years of brain aging (33).

In summary, assessing adipose tissue distribution (particularly VAT accumulation via VFA or surrogate indicators) can serve as a valuable predictive tool for metabolic disease risk and adverse outcomes, providing critical guidance for personalized clinical decision-making and optimal treatment strategy formulation.

## 3 Methods for assessing VAT

Various methods are available for obesity assessment, including BMI, Waist Circumference (WC), Waist-to-Hip Ratio (WHR), Waist-to-Height Ratio (WHtR), body fat percentage, and VFA. Among these, BMI is clinically widespread due to its convenience and low cost. However, it has inherent limitations, as it cannot distinguish between fat mass and lean body mass (34). Compared with BMI, anthropometric indicators such as WC, WHR, and WHtR are more effective predictors of central obesity (35). In 2024, the European Obesity Research Association proposed that WHtR ≥ 0.5 combined with BMI ≥ 25 kg/m^2^ as a new diagnostic standard for obesity (24). Nevertheless, these anthropometric measures cannot differentiate between intra-abdominal (visceral) and subcutaneous fat distribution, limiting their ability to predict disease risks (36).

Given this critical limitation of conventional anthropometric indices in discerning VAT, there has been increasing interest in developing and validating more sophisticated indices that aim to better quantify VAT distribution and its metabolic function, moving beyond simple anatomical measurements. For a comparative overview of various VAT assessment methods, including their principles, advantages, and disadvantages (e.g., imaging techniques like CT/MRI/DEXA and surrogate indices like VAI and Bioelectrical Impedance Analysis [BIA]), please refer to Table 1.

**Table 1:** Comparison of VAT measurement methods and their advantages and disadvantages.

Comparison of VAT measurement methods				
Measurement methods	Brief introduction	Diagnostic criteria/methodology	Benefits	Drawbacks
VAI	- Comprising anthropometry and lipid indicators; - It serves as agender-specific marker for evaluating VAT distribution and function.	- VAI score calculation formula (118): Males: VAI = [WC (cm)/39.68 + 1.88 × BMI (kg/m^2^)] × [TG (mmol/L) /1.03] × [1.31/HDL-C (mmol/L)]; Females: VAI = [WC (cm) /36.58 + 1.89 × BMI (kg/m^2^)] × [TG (mmol/L) /0.81] × [1.52/HDL-C (mmol/L)] - The VAI score offers an estimate of visceral obesity, where a higher score signifies a greater estimated VAT accumulation (37).	- The calculation is simple; - Data is easily obtainable; - The influence of gender on human fat distribution is taken into account.	- It may not be possible to fully capture the VAT distribution of all individuals. - For populations of different ethnicities, adjustments may need to be made to the formula to enhance accuracy.
BIA	A safe current is passed through the subject’s body, measuring the current flow from the wrist to the ankle, allowing for an estimation of the overall body fat percentage.	The body fat (BF) for percent (%BF) cutoff is determining obesity versus overweight (119): - Overweight (BMI > 25 kg/m2): men: 25% BF, women: 36% BF; - Obesity (BMI > 30 kg/m2): men: 30% BF, women: 42% BF.	- Quick and easy to use; - Portable; - Relatively inexpensive; - Accurate measurement of total fat content.	Not suitable for measurement of localized fat content due to a variety of factors such as race, gender, medication and device-specific differences.
DEXA	DEXA employs X-ray beams to assess three key body compositions: fat content and distribution, lean body mass, and bone density.	Estimation of total and regional body fat and lean tissue mass	- Accurate; - Precise; - Clinically available - Provides information on overall and regional adipose tissue percentages.	Very low radiation exposure: Standard equipment may not be suitable for severely obese individuals.
CT	CT uses x-ray to perform tomographic scanning on the human body. Through computer processing, it generates high-resolution images of the body’s transverse, coronal, or sagittal planes, facilitating the analysis of bod-y composition.	CT and MRI are the gold standard for measuring VFA (120): - Normal: VFA < 80 cm^2^; - central obesity: VFA ≥ 80 cm^2^.	- Highly accurate; - Regional analysis was used to assess fat-free mass for SAT and VAT.	- Expensive; - High levels of ionizing radiation, requires specialized software and knowledge for image analysis; - Standard equipment cannot accommodate morbidly obese individuals; - Time-consuming; - Complex. - CT scans are not suitable for infants, young children, or pregnant women.
MRI	MRI utilizes radio frequency signals, which are produced by the interaction between tissue protons and magnetic fields, to create images for assessing body adipose tissue.		- Highly accurate; - Quantifies body composition at the tissue-organ level; - Provides direct measurement of tissue cross-sectional area; - Radiation-free; - Unlimited number of tests.	- Expensive instruments; - High inspection costs; - Difficult to utilize in large sample clinical trials; - Requires analysis software and expertise to quantify body composition in images; - Exams are long and loud and can reduce subject compliance.

SAT, subcutaneous adipose tissue; VAT, visceral adipose tissue; VFA, Visceral fat area; WC, waist Circumference; TG, triglycerides; HDL-C, High-Density Lipoprotein Cholesterol; BIA, Bioelectrical Impedance Analysis; DEXA, Dual-energy X-ray Absorptiometry; BMI, body mass index; VAI, Visceral Adiposity Index.

### 3.1 VAI

VAI is a specific indicator designed to evaluate VAT distribution and function. Unlike simple anthropometric measures, it integrates BMI, WC, and gender, while also accounting for the effects of triglycerides (TG) and high-density lipoprotein cholesterol (HDL-C) on fat distribution and metabolic function (37). VAI exhibits a strong correlation with VAT, insulin sensitivity, and impaired glucose metabolism (38), and is significantly associated with heart failure (39) and metabolic syndrome (40).

The Tehran Lipid and Blood Glucose Study is a cohort study involving 9,645 participants, including 1,458 patients aged ≥ 21 years with prediabetes, and the average follow-up time is 9 years. The results show that adipose tissue dysfunction assessed by VAI can be used as a reliable marker of VAT metabolism and can predict the future blood glucose status of patients with prediabetes (41). In a study of 1,241 patients with T2DM, VAI and SAT were measured to explore correlations between obesity indices and DKD. Results showed that the urinary albumin-to-creatinine ratio (UACR) was positively correlated with VAI, indicating the utility of VAI in assessing DKD severity and predicting DKD development risk (42). Similarly, another study confirmed a positive correlation between VAI levels and proteinuria severity (43).

VAI offers advantages such as simple calculation, easy data acquisition, and no radiation exposure. However, it has limitations: it may not fully capture VAT distribution in all individuals—particularly those with normal BMI but high visceral adiposity (normal – weight obesity). Additionally, VAI was initially developed in the Italian population, and ethnic differences in adipose tissue distribution (e.g., between Europeans and East Asians) (44) mean it may not accurately reflect VAT in Chinese adults.

### 3.2 Chinese visceral adiposity index

To address the limitations of traditional VAI in Chinese populations, researchers developed the CVAI. Based on the original VAI, CVAI further incorporates age as a variable and adjusts for computed tomography (CT) values (a marker of adipose tissue density, reflecting fat quality) (45), aiming to better characterize visceral adiposity distribution and metabolism in Chinese individuals. Compared with conventional obesity assessment indices, CVAI has demonstrated higher accuracy in multiple studies, including predicting T2DM (46), cardiovascular event risk (47), metabolic syndrome (48), and new-onset stroke risk (49).

## 4 Relationship between VAT and DKD

### 4.1 Association between systemic VAT indicators and DKD

Systemic VAT assessment primarily relies on VFA and VAI. Both indicators correlate with DKD pathogenesis and progression but differ in clinical utility.

#### 4.1.1 VFA and DKD

VFA exhibits a stronger correlation with microalbuminuria than SAT (50), and an inverse relationship with glomerular filtration rate—increased visceral adiposity is accompanied by reduced renal filtration capacity (51). A meta-analysis exploring the association between central obesity parameters (VFA, WC, WHR, WHtR) and DKD in patients with T2DM confirmed that VFA has a strong, independent association with elevated UACR and a higher DKD incidence (52). It evaluates the association between central obesity and DKD comprehensively and reasonably, and makes up for the limitations of previous meta-analysis, such as the non-differential misclassification of albuminuria and CKD status, which leads to the conclusion that central obesity patients are not related to DKD (53).

A cross-sectional study from the National Health and Nutrition Examination Survey (NHANES), including 2,965 subjects (2,706 without albuminuria), divided participants into three VFA groups: low (0–60 cm^2^), moderate (60–120 cm^2^), and high (≥120 cm^2^). Results showed that high VFA was an independent risk factor for albuminuria, and the correlation between diabetes and albuminuria was strengthened with increasing visceral adiposity (54).

Chinese population studies have yielded consistent findings: T2DM patients with visceral obesity (VFA ≥ 100 cm^2^) had significantly higher UACR than non-viscerally obese patients, and VFA was strongly positively correlated with UACR (55). A retrospective cohort study further demonstrated that higher VFA was associated with increased DKD incidence, alongside elevated blood creatinine, urinary microalbumin, and UACR, and decreased estimated glomerular filtration rate (eGFR) (56).

These findings suggest that VFA may mediate the association between metabolic factors and renal outcomes. Quantitative VFA assessment is thus a promising biomarker for early detection of DKD, providing clinicians with a tool for timely intervention in DKD management.

#### 4.1.2 VAI and DKD

VAI is a robust metabolic predictor with well-defined clinical applications. It has been confirmed to predict diabetes development (46, 57), with gender-specific utility—its predictive accuracy is superior in females (Area Under the Curve = 0.82) (58).

Previous studies have mostly studied the relationship between VAI and diabetes mellitus, cardiovascular and cerebrovascular risk, and rarely studied the effect of VAI on diabetic renal complications. Subsequent horizontal and longitudinal studies have shown that VAI is associated with renal pathophysiological changes, and may be a simple and cost-effective indicator for predicting DKD (59–61), which has independent predictive value for compound renal outcomes (including albuminuria progression and eGFR decline) in diabetic patients (11). Collectively, VAI serves as a dual-purpose biomarker, enabling simultaneous evaluation of metabolic risk and renal impairment in at-risk populations.

### 4.2 Effect of renal ectopic adipose tissue deposition on DKD

Renal ectopic adipose tissue, mainly including PRAT and renal sinus fat (RSF), is anatomically adjacent to the kidneys and exerts direct effects on renal function. Unlike systemic VAT, these local depots may play a more immediate role in DKD pathogenesis.

#### 4.2.1 PRAT and DKD

PRAT is a unique subtype of VAT distributed around the kidneys, with anatomical and metabolic characteristics distinct from other VAT depots. It participates in energy metabolism, tissue differentiation, and immune regulation, and is closely associated with renal and systemic metabolic health (9). The unique anatomical location of PRAT and the developmental heterogeneity of its precursor cells may contribute to its strong association with CKD progression (62).

A cross-sectional study of 171 T2DM patients found that increased ultrasound-measured PRAT thickness was independently inversely correlated with eGFR, with gender differences: males with the same WC had a more pronounced inverse correlation between PRAT and eGFR, suggesting PRAT’s role in renal dysfunction in T2DM (63). This is consistent with another study (62), which showed that CT-measured PRAT remained strongly independently associated with eGFR after adjusting for confounding factors (e.g., systemic VAT). Longitudinal analysis further confirmed that PRAT independently predicts CKD incidence in T2DM patients, outperforming whole-body, subcutaneous, and systemic VAT.

Receiver Operating Characteristic (ROC) curve analysis revealed that PRAT has predictive value only when eGFR < 60 mL/min/1.73 m^2^ (*p* < 0.05), with an optimal cutoff of 13.65 mm (64). Additionally, a large-scale study of 959 T2DM patients found that a PRAT threshold of 0.90 cm reliably predicts albuminuria onset (both cross-sectionally and longitudinally), while a higher cutoff of 1.56 cm may predict DKD progression (65).

#### 4.2.2 RSF and DKD

RSF is an ectopic perivascular visceral adipose depot located in the renal sinus region, anatomically enveloping the renal vasculature, lymphatic network, and calyceal system. It is spatially adjacent to the adventitia of renal arteries (of all calibers) and has unique functional properties. As RSF accumulates, it can compress renal blood vessels (66), leading to hypertension, increased cardiovascular risk, and impaired renal function.

##### 4.2.2.1 Association with DKD pathogenesis

A retrospective MRI analysis of 126 subjects showed that bilateral RSF volume was significantly correlated with BMI, VAT area, hepatic fat fraction, and pancreatic fat fraction (67). A cross-sectional study further identified RSF as a pathogenic mediator of obesity-related nephropathy, contributing to renal parenchymal injury (68). For T2DM patients without overt CKD, elevated RSF volume is independently associated with reduced GFR and increased renal vascular resistance, suggesting it may accelerate CKD progression via hemodynamic and microvascular dysregulation (69). Similarly, intrarenal parenchymal fat (RIPF) is associated with lower eGFR and its decline, but its causal role in DKD remains unclear (70).

Notably, RSF accumulation precedes overt glycemic dysfunction. An MRI study comparing 230 normoglycemic controls, 87 prediabetic, and 49 diabetic individuals found progressive enlargement of renal volume, sinus dimensions, and RSF deposition along the glycemic continuum—prediabetic subjects had significantly more RSF than normoglycemic controls (*p* < 0.01) (71).

##### 4.2.2.2 Value in early DKD diagnosis

RSF has potential as a marker of early subclinical renal injury. A clinical study of T2DM patients documented a positive correlation between RSF volume and UACR (standardized *β* = 0.27, *p* = 0.016), alongside an association with elevated HbA1c. Among 95 enrolled patients, 87 had UACR < 30 mg/g, highlighting RSF’s utility in identifying early-stage DKD (72). Another study comparing 29 controls, 27 T2DM non-CKD patients, and 48 T2DM early CKD patients (UACR ≥ 30 mg/g) found that RSF volume was higher in T2DM patients with early CKD, suggesting it may be a causative factor for CKD in T2DM (72).

##### 4.2.2.3 Current research gaps

The clinical translation of RSF metrics is limited by a scarcity of longitudinal cohort studies and interventional trials. To establish RSF volumetry as a credible early biomarker for DKD progression, large-scale, multicenter studies are urgently needed.

## 5 Potential mechanisms by which VAT affects DKD

**Figure 1:**
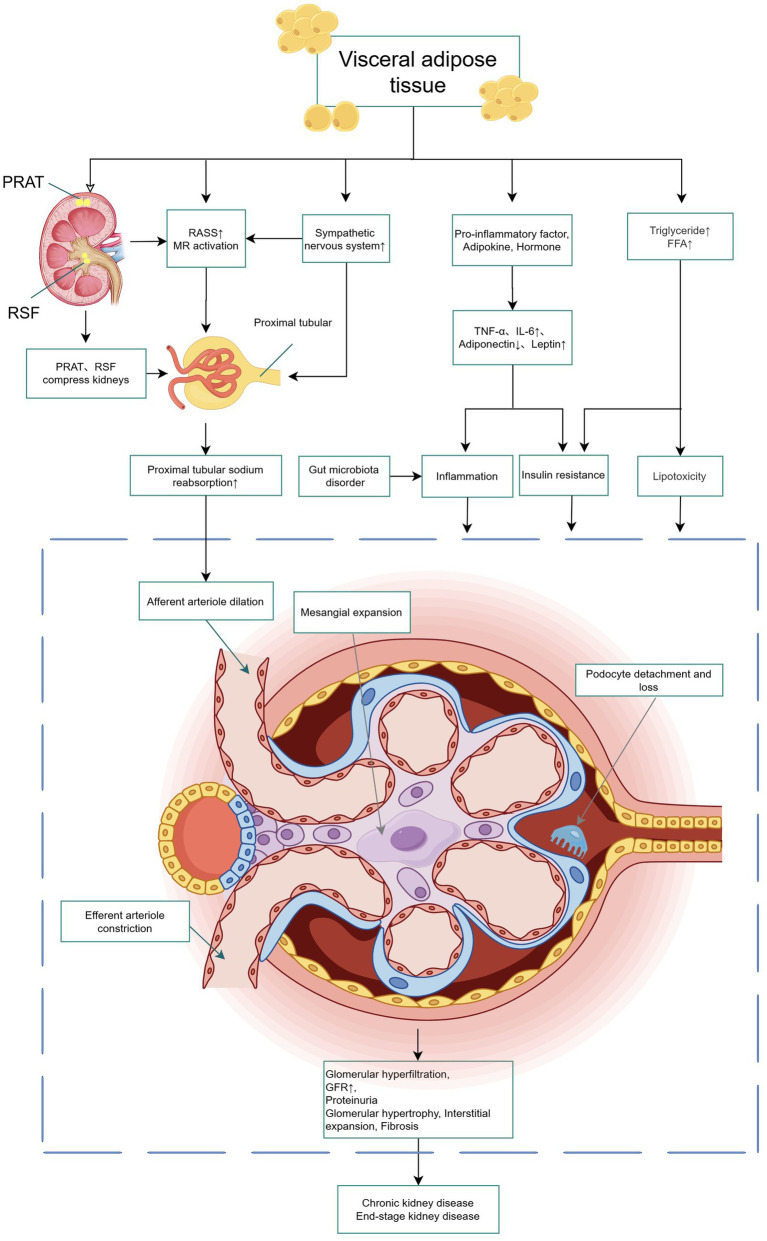
Schematic representation of how VAT drives DKD. VAT, along with RSF and PRAT, contributes to renal injury via multiple mechanisms, encompassing hemodynamic stress resulting from alterations in arteriolar tone, metabolic disturbances induced by insulin resistance and lipotoxicity, and chronic inflammation triggered by pro-inflammatory adipokines. When these processes are coupled with physical compression exerted on the renal structure, they can precipitate glomerular hyperfiltration, fibrosis, a progressive decline in renal function, and ultimately culminate in chronic kidney disease. PRAT, perirenal adipose tissue; RSF, renal sinus fat; RAAS, renin-angiotensin system; MR, mineralocorticoid receptor; FFA, free fatty acid; TNF-α, Tumor Necrosis Factor-α; IL-6, interleukin-6; GFR, glomerular filtration rate.

Although the underlying mechanisms linking VAT to DKD remain incompletely understood, cumulative evidence supports a multi-pathway regulatory network. These mechanisms primarily include hemodynamic dysregulation mediated by neuroendocrine activation, and metabolic and inflammatory disorders driven by adipose tissue dysfunction. Notably, renal ectopic fat deposition further exacerbates these pathological processes. The synergistic action of these mechanisms in promoting DKD progression is illustrated in Figure 1.

### 5.1 Hemodynamic dysregulation mediated by renin-angiotensin-aldosterone system activation

Obesity-induced renal hemodynamic disorders primarily manifest as glomerular hyperperfusion and hyperfiltration, which are closely associated with activation of RAAS. VAT serves as an important endocrine organ that expresses and secretes angiotensinogen, whose levels increase in parallel with BMI and leptin concentrations (73). Additionally, adipose tissue-derived factors such as leptin and mineralocorticoids can trigger RAAS activation, leading to elevated levels of angiotensin II (Ang II) and aldosterone.

Ang II exerts dual effects on renal hemodynamics: it dilates afferent arterioles and constricts efferent arterioles, resulting in glomerular hypertension that exacerbates glomerular injury and promotes glomerulosclerosis (73). Meanwhile, Ang II activates sodium channels in renal tubular epithelial cells, enhancing sodium reabsorption in both proximal and distal tubules. Adipocytes also secrete aldosterone-releasing factors (e.g., angiotensin II, catecholamines), which stimulate the adrenal cortex to synthesize and secrete aldosterone, further promoting renal sodium and water reabsorption. Glomerular hypertension and water-sodium retention collectively increase renal blood flow, inducing mechanical damage to podocytes. As key components of the glomerular filtration barrier, podocyte loss or dysfunction impairs barrier integrity, ultimately leading to proteinuria (73). Moreover, leptin can exacerbate proteinuria by activating the sympathetic nervous system and enhancing renal sodium reabsorption (74).

### 5.2 Metabolic and inflammatory disorders driven by adipose tissue dysfunction

VAT pathological expansion disrupts its endocrine and metabolic homeostasis, leading to abnormal secretion of adipokines, free fatty acids (FFAs), and pro-inflammatory mediators. These factors collectively induce insulin resistance, lipotoxicity, and chronic inflammation, which synergistically promote DKD progression.

#### 5.2.1 Insulin resistance (IR)-mediated renal injury

IR, characterized by reduced target tissue sensitivity to insulin and consequent uncontrolled hyperglycemia, is a key link between VAT accumulation and DKD. Pathological VAT expansion impairs its endocrine function, leading to aberrant secretion of lipotoxic mediators (e.g., FFAs) and adipocytokines: leptin levels are elevated (75), adiponectin levels are reduced, and pro-inflammatory cytokines (tumor necrosis factor-alpha [TNF-*α*], interleukin-6 [IL-6]) are upregulated. These factors collectively inhibit insulin signaling pathways (76).

Traditional perspectives attribute obesity-induced IR primarily to defects in insulin receptor substrate (IRS) phosphorylation, while emerging evidence identifies sympathetic nervous system overactivation as a central regulator of adipose metabolic dysfunction and systemic IR (77). Clinically, IR is closely associated with DKD hallmarks: glomerular hyperfiltration, albuminuria, and progressive renal function decline (78). Additionally, sustained elevation of circulating FFAs (resulting from increased VAT lipolysis) induces pancreatic *β*-cell lipotoxicity via endoplasmic reticulum stress-mediated apoptosis and impaired glucose-stimulated insulin secretion (79), further exacerbating hyperglycemia and renal damage. Ectopic lipid deposition in non-adipose tissues (including the kidney) induced by excessive FFAs also disrupts local insulin signaling (80), forming a “metabolic vicious cycle” in DKD progression.

#### 5.2.2 Lipotoxicity-induced renal parenchymal damage

Lipotoxicity refers to the abnormal accumulation of lipids in non-adipose tissues, leading to cellular dysfunction, damage, or death. Dyslipidemia promotes renal lipid deposition through dysregulation of lipid uptake, synthesis, fatty acid oxidation, and export, ultimately contributing to acute kidney injury and chronic kidney disease (81, 82). In the kidney, lipotoxicity primarily targets podocytes and renal tubular epithelial cells, mediating damage via multiple pathways: mitochondrial dysfunction, endoplasmic reticulum stress, excessive reactive oxygen species (ROS) production, and inflammatory stress (83).

Abnormal metabolism of lipids such as cholesterol, triglycerides, fatty acids, and phospholipids further accelerates renal fibrosis progression (84, 85). Specifically, lipid accumulation in podocytes leads to loss and dysfunction, while renal tubular epithelial cell damage triggers interstitial fibrosis, ultimately impairing the glomerular filtration barrier and inducing tubular lesions (86). Recent studies have identified Dock5 as a key regulatory molecule in podocyte lipotoxicity: Dock5 deficiency exacerbates podocyte damage in proteinuria-associated nephropathies by inhibiting Rac1 GTPase activation and disrupting cytoskeletal stability, suggesting it may be a potential therapeutic target (87).

#### 5.2.3 Chronic inflammation and gut microbiota dysbiosis

Chronic inflammation is a core pathological feature of both VAT accumulation and DKD, with renal interstitial inflammation and fibrosis being prominent manifestations of DKD progression (88). VAT hypertrophy drives two key inflammatory cascades:

1. Adipose-derived inflammatory mediators: Hypertrophied adipocytes secrete pro-inflammatory adipokines (leptin, resistin) and recruit M1-type macrophages, which secrete cytokines such as TNF-*α* and IL-6 (89). These factors induce systemic inflammation, increase renal vascular endothelial permeability, and enhance sympathetic nervous activity (90). Additionally, TNF-α and IL-6 inhibit phosphorylation of insulin receptors and downstream signaling molecules (e.g., IRS-1), impairing intracellular insulin signal transduction and further exacerbating IR (91, 92).
2. Gut microbiota-inflammation-renal axis: Obesity-induced gut dysbiosis contributes to systemic inflammation via multiple pathways. Obese microbiota exhibits impaired ethanolamine catabolism, leading to ethanolamine accumulation that upregulates miR-101a-3p, downregulates the tight junction protein zonula occludens-1, and increases intestinal permeability (93). A population-based study identified gut microbiota-driven neutrophil infiltration in VAT of obese individuals, accompanied by elevated TNF-*α* and IL-6 levels in VAT (94), forming a “VAT-gut inflammation loop” that promotes DKD. Moreover, gut microbiota disruption can induce renal tubulointerstitial inflammation via release of bacterial outer membrane vesicles (OMVs, which carry lipopolysaccharides and other toxic components), directly mediating renal injury (95).

Notably, reduced levels of anti-inflammatory adipokines (e.g., adiponectin) further exacerbate oxidative stress and inflammatory responses, accelerating renal interstitial fibrosis and glomerular damage (84, 86). Collectively, targeting the VAT-inflammatory-gut microbiota axis may represent a novel therapeutic strategy for DKD.

## 6 Mechanisms of perirenal and RSF in renal injury

Ectopic adipose tissue deposition in the renal region—primarily RSF and PRAT—exerts direct and indirect damaging effects on renal structure and function. Both depots share common pathogenic pathways (mechanical compression and inflammatory mediator secretion) but exhibit distinct characteristics due to their anatomical locations.

### 6.1 Mechanism of renal injury caused by RSF

RSF is a depot of ectopic perivascular VAT localized within the renal sinus, surrounding the renal vasculature (including hilar arteries, veins, and microvessels), lymphatic networks, and pelvicalyceal system (96). Its unique anatomical positioning enables two key pathogenic mechanisms.

#### 6.1.1 Hemodynamic disruption via mechanical compression

RSF accumulation directly compresses hilar vascular structures and intrarenal microvessels, leading to elevated renal interstitial pressure and intrarenal venous pressure (69). This hemodynamic perturbation triggers two cascades: first, it activates RAAS, promoting renal sodium retention and disrupting tubuloglomerular feedback; second, it reduces renal blood flow and glomerular perfusion, exacerbating hyperfiltration-induced nephron injury (e.g., podocyte loss and glomerulosclerosis).

#### 6.1.2 Inflammatory and fibrotic microenvironment induction

RSF functions as an endocrine-active perivascular tissue, secreting pro-inflammatory adipokines (leptin, resistin), monocyte chemoattractant protein-1 (MCP-1), and cytokines (IL-6) (97). These mediators drive renal injury through multiple pathways: MCP-1 specifically recruits circulating monocytes to differentiate into M1-type pro-inflammatory macrophages in the renal parenchyma (98), amplifying local inflammatory responses; leptin and IL-6 induce hypoxia in renal tissues and enhance oxidative stress, further activating inflammatory signaling cascades. Notably, RSF accumulation and inflammation form a positive feedback loop—elevated MCP-1 expression in RSF promotes macrophage infiltration, which in turn secretes more cytokines to accelerate RSF expansion and renal fibrosis (97, 98).

### 6.2 Mechanism of renal injury induced by PRAT

PRAT is a fat pad surrounding the kidneys, located between the renal fibrous capsule and retroperitoneal fascia, and is a specialized subtype of VAT (99). Compared with RSF, PRAT exerts more prominent effects through paracrine signaling and indirect compression:

#### 6.2.1 Renal parenchymal damage via mechanical compression

Increased PRAT thickness compresses the renal parenchyma and perirenal blood vessels, elevating intrarenal pressure while reducing intravascular blood flow (100). This compression impairs renal tubular reabsorption function and disrupts glomerular filtration dynamics, leading to sodium and water retention and subsequent renal interstitial edema—a process that exacerbates renal injury in diabetic patients.

#### 6.2.2 Paracrine-mediated metabolic and inflammatory injury

PRAT exhibits higher activity in adipokine synthesis and pro-inflammatory cytokine secretion than other visceral adipose depots, producing leptin, TNF-*α*, IL-6, and lipid metabolites (e.g., triglycerides, malondialdehyde) (90). These factors contribute to DKD progression through two key mechanisms: first, adipokine dysregulation (e.g., elevated leptin, reduced adiponectin) impairs insulin signaling and induces insulin resistance in renal tubular epithelial cells; second, pro-inflammatory cytokines and toxic lipid metabolites directly damage renal parenchymal cells (podocytes, tubular epithelial cells) and activate the TGF-β1/Smad pathway, promoting renal interstitial fibrosis (90, 100).

## 7 Targets for the treatment of VAT

As VAT drives renal impairment and DKD progression via multiple pathways, early intervention targeting VAT accumulation is critical for preventing or reversing renal damage. Currently, two classes of novel hypoglycemic agents—glucagon-like peptide-1 receptor agonists (GLP-1RAs) and sodium-glucose cotransporter 2 inhibitors (SGLT2is)—have demonstrated potent effects on reducing VAT and conferring renoprotection, emerging as key therapeutic targets.

### 7.1 GLP-1RAs

GLP-1RAs integrate hypoglycemic, weight-reducing, cardiovascular-protective, and renoprotective effects, reducing the risk of cardiovascular events and DKD progression (101–103). Their ability to modulate fat distribution has gained increasing attention in recent years.

#### 7.1.1 Effects on systemic and abdominal VAT

While GLP-1RAs have long been known to reduce body weight (104), the first systematic meta-analysis (2022) specifically evaluating their impact on fat distribution confirmed that liraglutide and exenatide (two classic GLP-1RAs) reduce both VAT and SAT in patients with T2DM (105). However, this analysis was limited to two agents, requiring further research to verify consistency across other GLP-1RAs.

A subsequent meta-analysis of semaglutide (a long-acting GLP-1RA) showed more precise effects: compared with the control group, the GLP-1RA group achieved an average fat mass reduction of 2.25 kg, with SAT and VAT decreasing by 38.35 cm^2^ and 14.61 cm^2^, respectively. Notably, the fat-lowering effect was more pronounced in patients with diabetes (106).

#### 7.1.2 Effects on renal ectopic fat

Growing evidence supports GLP-1RAs’ ability to target renal-localized ectopic fat. A study using ultrasound to assess abdominal fat pools confirmed that liraglutide treatment significantly reduced PRAT (107). Another MRI-based study explored liraglutide’s effects on RSF and potential racial differences, finding that Western European patients treated with liraglutide showed slower RSF accumulation—suggesting possible ethnic variations in RSF dynamics with glucose-modulating therapy (108). However, this study had limitations: a small sample size, broader inclusion criteria for the South Asian group versus the Western European group, and results requiring validation in larger cohorts.

#### 7.1.3 Mechanisms linking fat reduction to renoprotection

The renoprotective effects of GLP-1RAs are partially mediated by VAT reduction. In a high-fat diet-induced obese CKD rodent model, liraglutide attenuated renal lipid accumulation and improved mitochondrial function via activating the Sirt1/AMPK/PGC1α signaling pathway, thereby mitigating obesity-related renal injury (109). MRI studies further confirmed that liraglutide reduces PRAT deposition, enhances renal cortical perfusion, and improves renal tissue oxygenation—with diminished cortical oxygenation being a known driver of renal fibrosis (110). These findings directly link GLP-1RA-induced VAT reduction to renal protection.

### 7.2 SGLT2is

SGLT2is also exhibit weight-reducing effects, with consistent evidence of VAT reduction (111, 112). Their mechanism of action on adipose tissue and renoprotection has been partially elucidated.

A study investigating dapagliflozin’s effects in obese T2DM mice found that the drug significantly upregulated the expression of browning-related genes (UCP-1, PGC-1α, CIDEA) in white adipose tissue (WAT). WAT browning converts energy-storing white adipocytes into energy-consuming beige adipocytes, thereby reducing fat accumulation. Additionally, dapagliflozin downregulated proinflammatory mediators in WAT, regulated cellular autophagy, improved local inflammation, and promoted angiogenesis (113). These findings provide a theoretical basis for SGLT2i use in obese T2DM patients, though further research is needed to validate these mechanisms in humans.

### 7.3 Common renoprotective value of GLP-1RAs and SGLT2is

Beyond their effects on fat and glucose control, GLP-1RAs and SGLT2is share robust renoprotective properties: they significantly reduce urinary protein excretion, attenuate renal inflammation (114), and delay eGFR decline in diabetic patients—regardless of baseline proteinuria severity (115). Clinical evidence shows these agents reduce CKD progression risk by 33% and slow the annual rate of renal function decline (116). Their nephroprotective mechanisms extend beyond hypoglycemia, with VAT reduction and subsequent mitigation of lipotoxicity, inflammation, and hemodynamic dysregulation being key contributors (101, 117). The comparative analysis of the studies cited in this paper is shown in Table 2.

**Table 2:** To summarize the literature cited in this article on clinical studies related to VAT and DKD.

Citation summarization					
First Author, Year (Ref.)	Study design	Sample size	Adjustment covariates	Outcomes and results	Limitations
Yufang Liu, 2024 (54)	Cross-sectional survey, observational	2,965 participants (2,706 without albuminuria).	Age, sex, race, smoking, and blood pressure.	High VFA may constitute an independent risk factor for albuminuria.	Failure to exclude type 1 diabetes may have influenced the results; Bias of DXA for VAT measurements.
ZENG Bing-bing, 2024 (55)	Cross-sectional survey, observational	169 patients with T2DM.	Age, LDL-C, BUN, Scr and PRAT.	A strong negative relationship has been identified between PRAT and eGFR in patients with T2DM.	Cannot determine causal relationship between PRAT thickening and decreased GFR in patients with T2DM; Single-center study; Relatively small sample size.
Xiangjun Chen, 2021 (62)	Longitudinal study, observational study	383 patients with T2DM.	Age, sex, diabetes duration, hypertension history, insulin therapy, antihypertensive drugs, HbA1c, eGFR, lipid-lowering drugs, SBP, TBF, SAT, and VAT, PRAT.	In patients with T2DM, perirenal fat is a better predictor of CKD than total, subcutaneous, or VAT.	The sample size was small, and the follow-up period was short. It was a single-center study; The effect of perirenal fat on CKD outcomes should be verified in different populations.
Yuan Fang, 2020 (63)	Cross-sectional survey, observational	171 patients with T2DM.	Age, diabetes duration, FPG, TC, LDL-C, HbA1c, and PRAT.	PRAT was independently and negatively correlated with eGFR, especially in men.	Ultrasound and BIA measured PRAT and PnFT and VFA, respectively, with lesser accuracy than CT and MRI.
Hongtu Hu 1, 2022 (65)	Cross-sectional survey, longitudinal study	959 patients with T2DM were divided into three groups: without albuminuria (UACR <30 mg/g, 636 cases), subjects with microalbuminuria (30 mg/g < UACR < 300 mg/g, 191 cases), and subjects with macroalbuminuria (UACR > 300 mg/g, 132cases).	HbA1C, age, urine creatinine, ALB, urea, SCr, UA, TG, LDL-C, eGFR, Hb, PT, duration of diabetes mellitus, and hypertension levels.	Elevated PRAT are an independent risk factor for proteinuria in patients with diabetes. Patients with thicker PRF have a poorer prognosis and are more likely to develop proteinuria.	Single-center study; Relatively small sample size; Racial selection bias.
Qin-He Zhang, 2023 (67)	Retrospective study	126 normal Chinese subjects (46 men and 80 women).	VAT	The RSF volumes and FF values of the bilateral kidneys were significantly correlated with BMI, VAT area, hepatic fat fraction, and pancreatic fat fraction in the overall subjects, but not with SAT area	Causality cannot be inferred; RSF is manually segmented and subject to error; Limit the generalization of the results to other racial population
Michelotti, Filippo C. 2025 (70)	Retrospective study	221 participants, including people with type 1 (*n* = 66), type 2 diabetes (*n* = 55) and participants with normoglycemia (*n* = 26).	Sex, age, BMI, MAP and SAT and VAT volumes.	Renal intra-parenchymal fat is linked to lower eGFR and its decline	A causal relationship between RIPF and DKD could not be established.
Mike Notohamiprodjo, 2020 (71)	Cross-sectional survey	366 participants who were normoglycemic (*N* = 230), prediabetic (*N* = 87), or diabetic (*N* = 49).	Age, sex, VAT, HDL, LDL, urine albumin, liver fat, GFR and hypertension	Renal volume and particularly RSF volume already increases significantly in prediabetic subjects and is significantly associated with VAT.	The MRI’s semi-automated algorithm did not satisfactory separate renal cortex and medulla. The effect of race could not be evaluated.
Ling Lin, Ilona A. Dekkers, 2021 (72)	Cross-sectional survey	146 participants, including 95 T2DM patients and 51 healthy controls.	Age, sex, ethnicity and T2DM.	A larger RSF volume is associated with high levels of glycosylated hemoglobin and metabolic risk factors, and is associated with an increased UACR in patients with T2DM.	Does not allow interpretation of causality of association between RSF accumulation and clinical features; No gold standard GFR.

VFA, visceral fat area; DXA, dual-energy X-ray Absorptiometry; SAT, subcutaneous adipose tissue; VAT, visceral adipose tissue; PRFT, perirenal fat thickness; PRAT, perirenal adipose tissue; FPG, Fasting Plasma Glucose; TC, Total Cholesterol; LDL-C, high-density lipoprotein cholesterol; HDL, High-Density Lipoprotein; HbA1c, Glycated Hemoglobin A1c; eGFR, estimated Glomerular Filtration Rate; BIA, bioelectrical impedance analysis; PnFT, paranephric fat thickness; CT, Computed Tomography; MRI, Magnetic Resonance Imaging; SBP, Systolic Blood Pressure; TBF, total body fat; ALB, serum albumin; SCr, blood creatinine; UA, blood uric acid; TG, triglycerides; Hb, hemoglobin; PT, prothrombin time; PRF, Perirenal fat; RSF, renal sinus fat; UACR, urinary albumin-to-creatinine ratio; BMI, body mass index; MAP, mean arterial pressure; RIPF, renal intra-parenchymal fat.

## 8 Conclusions and prospects

In summary, accumulating evidence confirms a close and causal association between VAT accumulation and DKD. Despite the existence of various convenient anthropometric and metabolic index evaluation methods for clinical screening, the VFA measured by imaging technology (CT/MRI) remains the gold standard.

VAT promotes the progression of DKD through a multi-channel pathophysiological network: systemic disturbances originating from VAT, including dysfunctional activation of the RAAS, insulin resistance, lipotoxicity, and chronic inflammation driven by adipose tissue, collaborate with local renal injury (mechanical compression and secretion of inflammatory mediators) caused by renal ectopic fat. In conclusion, this VAT centric network accelerates the transition from early DKD to irreversible glomerulosclerosis and tubulointerstitial fibrosis.

The current clinical diagnosis of DKD primarily relies on eGFR and UACR. However, they exhibit significant limitations in early detection, as these indicators often remain normal even when subclinical renal pathological changes, such as increased glomerular filtration rate and ectopic fat deposition in the PRAT and RSF, have already occurred. This diagnostic delay poses a critical clinical challenge, given the potential renal-protective effects of early intervention. To address this unmet need, we propose that VAT, quantified by VFA, can serve as a robust and complementary early warning indicator for eGFR/UACR. The crucial aspect is that pathological changes driven by VAT emerge prior to the onset of proteinuria and eGFR decline, presenting a feasible clinical window for timely intervention.

Notably, renal ectopic fat, as a localized manifestation of visceral adiposity in DKD, has demonstrated potential pathogenic roles in renal hemodynamic disturbance and parenchymal injury, though its causal relationship with DKD progression requires further validation. Looking forward, key areas for future research include: (1) conducting large-scale prospective cohort studies to determine the optimal VFA/ectopic fat thresholds for predicting DKD in different ethnic groups (e.g., East Asians vs. Western populations); (2) exploring the combined predictive value of VFA, renal ectopic fat, and metabolic indices (e.g., CVAI) for DKD progression; and (3) validating whether targeting VAT (via GLP-1RAs, SGLT2is, or lifestyle interventions) can effectively delay early DKD onset. Advancing these studies will transition VAT assessment from theory to practice, bridge the critical early diagnosis gap, and pave the way for novel prevention strategies.
